# Visual acuity and retinal thickness outcomes following anti-VEGF therapy in RVO: influence of early vs. delayed treatment

**DOI:** 10.1186/s12886-026-04667-2

**Published:** 2026-03-14

**Authors:** Lexzion Chung, Blazej Staniszewski, Paul Johnstone

**Affiliations:** National Health Service Tayside, Dundee, UK

**Keywords:** Retinal vein occlusion, Anti-vascular endothelial growth factor (Anti-VEGF), Treatment timing, Visual acuity, Central retinal thickness

## Abstract

**Background/Aims:**

Retinal vein occlusion (RVO) is a common cause of vision loss, treated primarily with intravitreal anti-VEGF injections. This study aimed to investigate how the timing of treatment initiation affects visual acuity and retinal thickness outcomes in patients with branch (BRVO) and central retinal vein occlusion (CRVO).

**Methods:**

A retrospective observational study was conducted involving 86 patients diagnosed with RVO (54 BRVO, 32 CRVO) and treated with anti-VEGF therapy at a single UK centre. Patients were stratified into early (≤ 28 days) and delayed (> 28 days) treatment groups based on the time from diagnosis to first injection. Visual acuity (ETDRS letters) and central retinal thickness (µm) were recorded at diagnosis, after three loading doses, and at one year. Paired t-tests were used to assess changes over time. Associations between treatment timing and outcomes were evaluated using Spearman’s rank correlation, while between-group comparisons were performed using Mann–Whitney U and chi-square tests.

**Results:**

BRVO patients had significant VA gains at three injections (mean + 13.2 letters, *p* < 0.001) and at one year (*p* = 0.0002), while CRVO patients showed a non-significant trend toward improvement (*p* = 0.081). CRT reductions were significant in both CRVO and BRVO at three injections and at one year (*p* < 0.001). Earlier treatment (≤ 28 days) was weakly correlated with greater CRT reduction in CRVO (*p* = 0.047), but no significant association was found between treatment timing and VA change or likelihood of > 15-letter improvement in either group.

**Conclusions:**

While early treatment led to short-term visual and anatomical gains, long-term visual outcomes at one year were not significantly associated with treatment timing, suggesting other clinical and individual factors contribute to final prognosis.

## Introduction

 Retinal vein occlusion (RVO), encompassing branch retinal vein occlusion (BRVO) and central retinal vein occlusion (CRVO), is among the most common retinal vascular conditions and a significant cause of visual morbidity worldwide [1, 2]. RVO leads to retinal haemorrhage, venous stasis, and macular oedema—the latter being the principal cause of vision loss in affected individuals [3].

The introduction of intravitreal anti-vascular endothelial growth factor (anti-VEGF) therapy has markedly improved visual and anatomical outcomes in patients with RVO-related macular oedema. Randomized controlled trials, such as the Ranibizumab for the Treatment of Macular Edema following Branch Retinal Vein Occlusion: Evaluation of Efficacy and Safety (BRAVO) and the Ranibizumab for the Treatment of Macular Edema after Central Retinal Vein Occlusion Study: Evaluation of Efficacy and Safety (CRUISE) studies, have demonstrated that early and sustained anti-VEGF treatment results in substantial visual acuity (VA) gains and reduction in central retinal thickness (CRT) [4–6]. These findings have informed clinical guidelines and expectations around prompt treatment initiation.

In the United Kingdom, the Royal College of Ophthalmologists (RCOphth) guidelines recommend that patients with suspected RVO should be seen and treated by an ophthalmologist within 2–4 weeks of presentation [7]. However, in real-world settings, systemic healthcare limitations, delayed referrals, and logistical barriers can lead to treatment delays beyond this window.

The 12-month results of the CRYSTAL study indicated that patients who initiated anti-VEGF treatment within three months of CRVO diagnosis experienced greater improvements in VA compared to those who began treatment after three months [8]. However, contrasting findings from other real-world studies have reported no significant association between the duration of macular oedema prior to treatment and subsequent visual outcomes [9]. This inconsistency highlights the ongoing uncertainty in the literature, particularly within routine clinical settings where delays in treatment are not uncommon. The variability in findings underscores the need for further investigation into the clinical significance of treatment timing in RVO management.

This study aims to investigate whether the timing of anti-VEGF initiation—specifically, treatment within 28 days versus after 28 days from diagnosis—affects VA and CRT outcomes in patients with BRVO and CRVO over a 12-month period. By analysing real-world data from a single tertiary ophthalmology centre, we seek to assess the clinical relevance of treatment delay and contribute evidence toward optimizing treatment pathways for RVO management.

## Methods

### Study design and setting

This was a retrospective, observational cohort study conducted at a tertiary ophthalmology centre in the United Kingdom. The study analysed all newly diagnosed cases of cystoid macular oedema secondary to BRVO and CRVO who commenced anti-VEGF therapy between January 1 and December 31, 2023. All patients underwent a loading regimen of anti-VEGF therapy, consisting of three intravitreal injections administered at four-week intervals over a three-month period. Following this initial course, patients were managed on a pro re nata (PRN) treatment protocol.

### Inclusion and exclusion criteria

Inclusion criteria comprised adult patients (≥ 18 years) with a new diagnosis of BRVO or CRVO confirmed by clinical examination and imaging (OCT and/or fluorescein angiography), who received anti-VEGF intravitreal injections, and had a minimum follow-up of one year. Patients were excluded if they had any other retinal pathology that could confound visual outcomes (e.g., diabetic retinopathy, age-related macular degeneration), prior anti-VEGF treatment in the affected eye, or incomplete clinical records.

### Data collection

Demographic and clinical data were extracted from electronic medical records, including type of RVO, date of diagnosis, date of first injection, anti-VEGF drug of choice, number of injections received within one year of diagnosis, and Early Treatment Diabetic Retinopathy Study (ETDRS) VA and CRT at baseline measured on Spectralis^®^ OCT (Heidelberg Engineering, Heidelberg, Germany), after three injections, and at one year.

Patients were categorized into two groups based on time to first injection:

- **Early treatment**: First anti-VEGF injection administered within ≤ 28 days from diagnosis.
- **Delayed treatment**: First injection administered > 28 days after diagnosis.

The 28-day threshold was chosen to reflect the RCOphth guidelines that patients with suspected RVO should be seen and treated by an ophthalmologist within 2–4 weeks of presentation [7]. This cutoff was intended to represent a clinically and service-relevant distinction between treatment delivered within recommended timelines and treatment delayed beyond this window.

### Outcome measures

The primary outcomes were changes in VA (ETDRS letters) and CRT at two time points: after three anti-VEGF injections (short-term outcome) and at one year from diagnosis (long-term outcome). Secondary outcomes included the proportion of patients achieving significant visual improvement (≥ 15 ETDRS letters), no significant change, or worsening in vision.

### Statistical analysis

Descriptive statistics were used to summarize patient characteristics and clinical outcomes. To compare early versus delayed treatment groups, Mann-Whitney U tests were used for continuous variables and Chi-square tests for categorical variables. Spearman’s rank correlation was employed to assess associations between time to treatment and clinical outcomes.

To evaluate the statistical significance of changes in VA and CRT within patients over time, paired t-tests were performed for three comparisons: (1) from diagnosis to after three injections, (2) from diagnosis to one year, and (3) from after three injections to one year. These were conducted separately for CRVO and BRVO subgroups. A p-value < 0.05 was considered statistically significant. All analyses were conducted using SPSS (IBM Corp.).

Cases with missing critical clinical data were excluded from the final analysis; no imputation was performed.

## Results

A total of 86 patients met the inclusion criteria for this study, comprising 54 with BRVO and 32 with CRVO. The average age at diagnosis was 79 years. Of the 86 patients included in the study, 37 were male and 49 were female.

The average time from referral to initial clinic assessment was 30.6 days, ranging from same-day assessment (0 days) to a maximum delay of 154 days. After excluding three cases where RVO was not detected on first presentation, the mean referral-to-assessment time reduced slightly to 28.5 days. Notably, 25 patients were seen more than 28 days after referral.

From the point of being listed for treatment, the average time to first anti-VEGF injection was 24.5 days, with 27 patients receiving their initial injection more than four weeks after listing (10 of these patients had CRVO and 17 of them had BRVO) (Table 1).

**Table 1:** Distribution of early vs. delayed treatment by retinal vein occlusion subtype

Retinal Vein Occlusion Subtype	Early Treatment (≤ 28 days), *n* (%)	Delayed Treatment (> 28 days), *n* (%)	Total
Central retinal vein Occlusion (CRVO)	22 (68.8%)	10 (31.2%)	32
Branch Retinal Vein Occlusion (BRVO)	37 (68.5%)	17 (31.5%)	54
Overall	**59 (68.6%)**	**27 (31.4%)**	86

The mean time from being listed to first injection was approximately 16.6 days in the early treatment group (≤ 28 days) and 41.8 days in the delayed treatment group (> 28 days). On average, patients received approximately 4.6 anti-VEGF injections within the first year, with the number of injections in 1 year ranging from 3 to 10. Fifty-three patients had additional injections beyond the initial three loading doses. All patients initiated treated with Aflibercept (Eylea). Five patients were switched to faricimab (Vabysmo^®^) after completing the three initial loading doses of aflibercept. One patient was switched to ranibizumab biosimilar (Ongavia^®^) after a single dose of aflibercept, and one patient was switched to ranibizumab (Lucentis^®^) after a single aflibercept injection. Additionally, one patient received four doses of aflibercept followed by one dose of ranibizumab and subsequently one dose of bevacizumab due to temporary unavailability of ranibizumab. Overall, treatment switching was infrequent and insufficient to justify subgroup analysis for each regime.

Baseline visual acuity was similar between early and delayed treatment groups within each RVO subtype. In BRVO, mean baseline VA was 50.8 ETDRS letters (range 0–90) in the early treatment group and 49.2 letters (range 0–80) in the delayed group. In CRVO, mean baseline VA was 40.2 letters (range 0–85) in the early group and 45.8 letters (range 0–75) in the delayed group.

Following three initial loading doses, 29 patients (33.7%) demonstrated a **≥ **15-letter improvement in VA, while 41 patients (47.7%) showed no significant improvement, and 16 patients (18.6%) experienced worsening of vision. At the one-year mark from diagnosis, 30 patients (35.7%) maintained or achieved a **≥ **15-letter gain, 34 (40.5%) had no significant change, and 20 (23.8%) experienced decline in VA of ≥ 15 letters.

In the CRVO group, the mean VA at diagnosis was 41.8 ± 29.4 ETDRS letters, improving to 50.3 ± 26.8 after three injections, but declining slightly to 42.0 ± 31.7 at one year. The mean CRT in this group reduced from 604.2 ± 244.5 μm at baseline to 235.4 ± 156.3 μm after three injections and slightly increased to 340.2 ± 258.4 μm at one year.

In the BRVO group, the mean VA improved from 50.2 ± 20.3 ETDRS letters at diagnosis to 63.4 ± 13.1 after loading doses, with a slight reduction to 61.3 ± 18.5 at one year. CRT in BRVO decreased from 547.5 ± 205.3 μm at diagnosis to 232.0 ± 127.6 μm after three injections and was 276.5 ± 136.7 μm at the one-year mark.

These data indicate an early anatomical and functional response to anti-VEGF therapy, with a tendency toward stabilization or mild regression over the longer term.

**Fig. 1:**
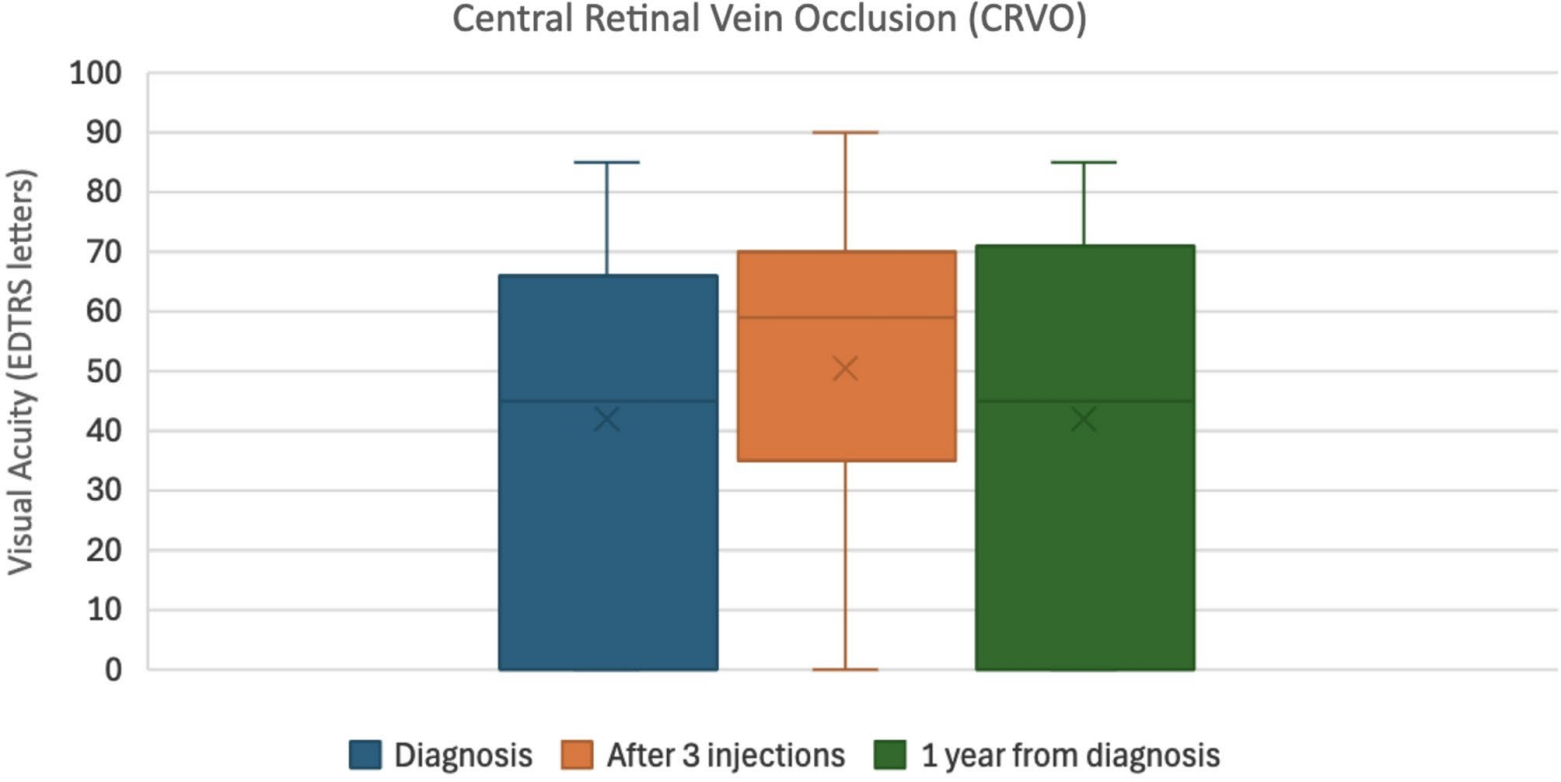
Box plot showing the median visual acuity in ETDRS letters and inter-quartile ranges at diagnosis, after 3 loading Anti-VEGF injections, and at 1 year from diagnosis for CRVO patients

**Fig. 2:**
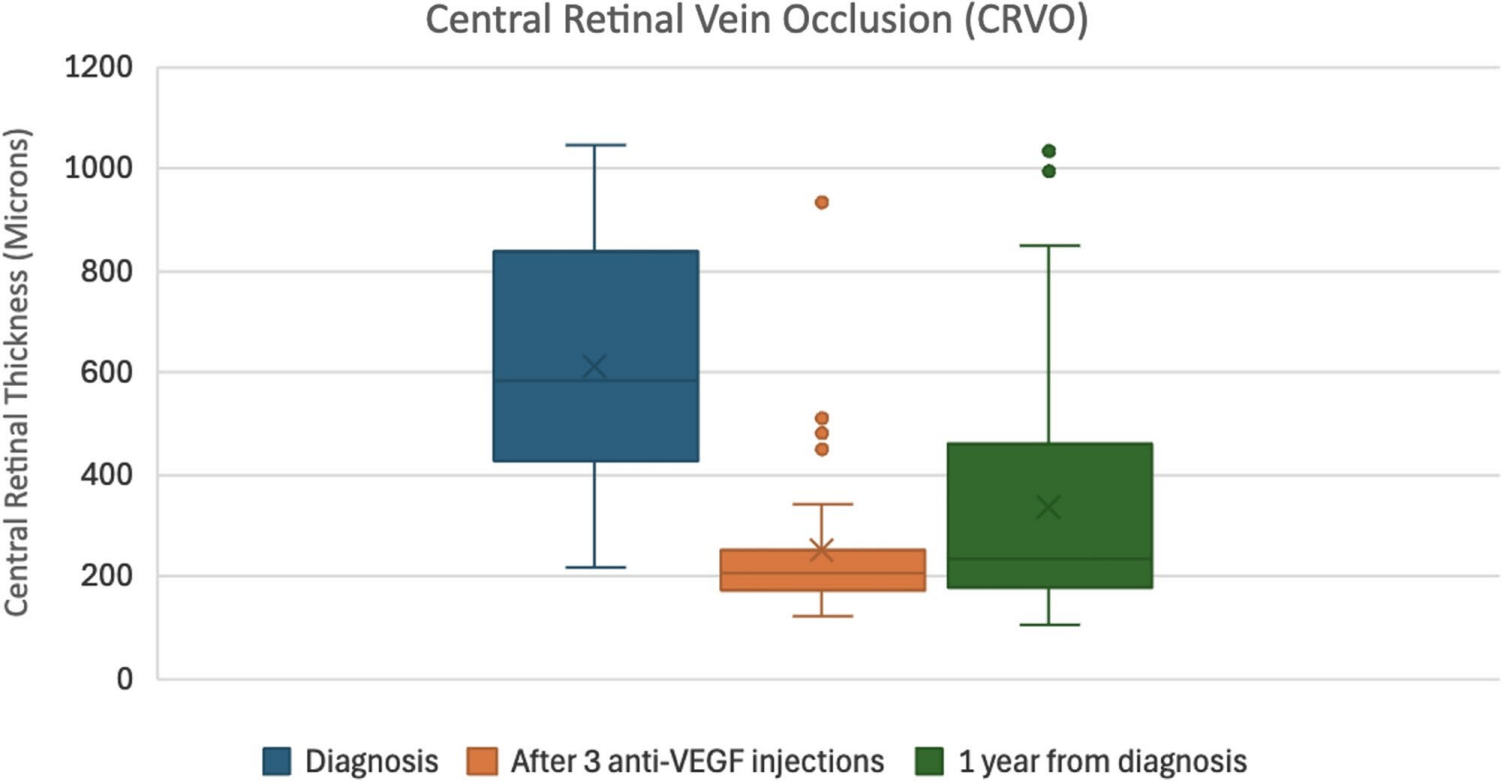
Box plot showing the median central retinal thickness in microns and inter-quartile ranges at diagnosis, after 3 loading Anti-VEGF injections, and at 1 year from diagnosis for CRVO patients

**Fig. 3:**
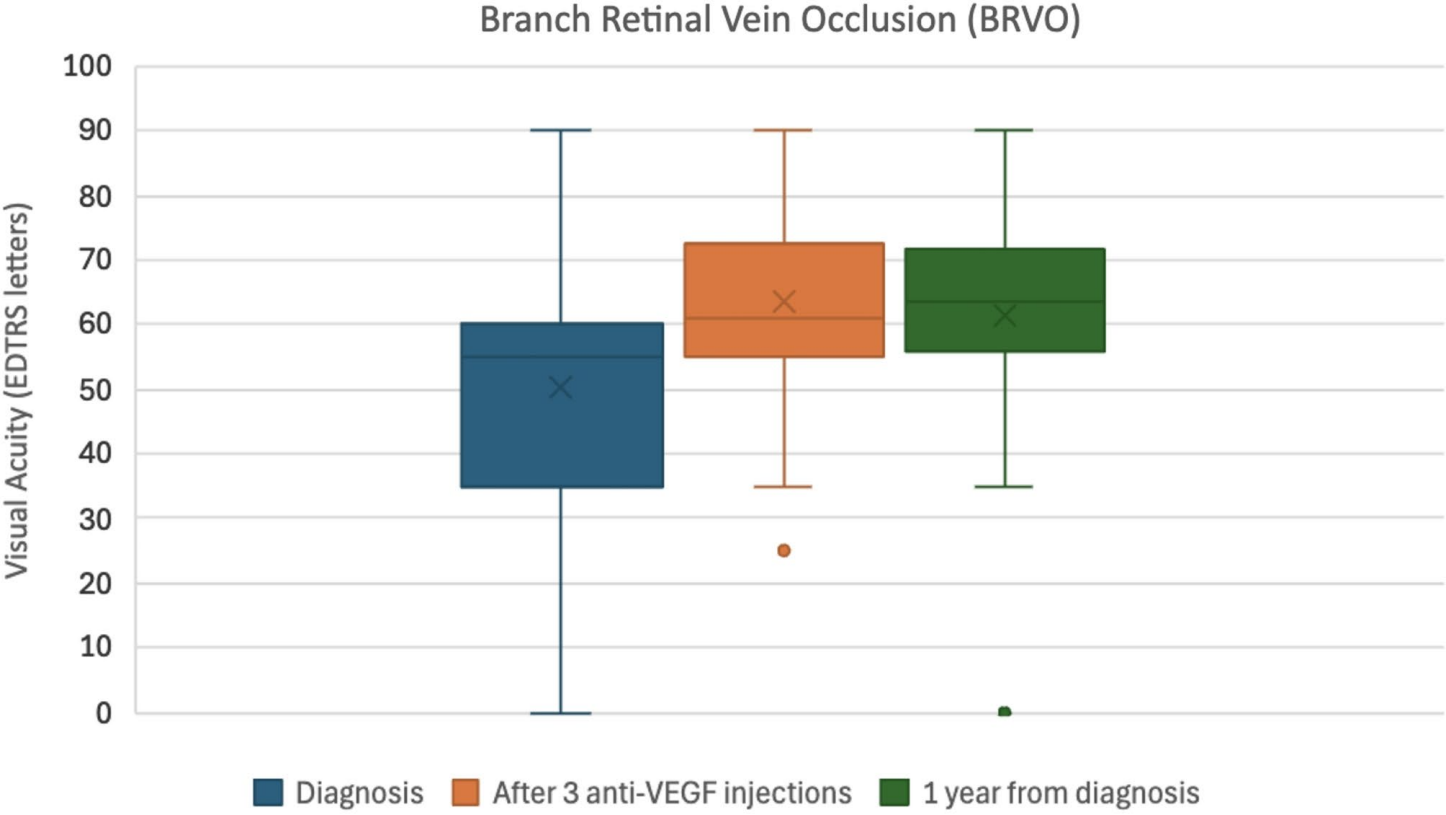
Box plot showing the median visual acuity in ETDRS letters and inter-quartile ranges at diagnosis, after 3 loading Anti-VEGF injections, and at 1 year from diagnosis for BRVO patients

**Fig. 4:**
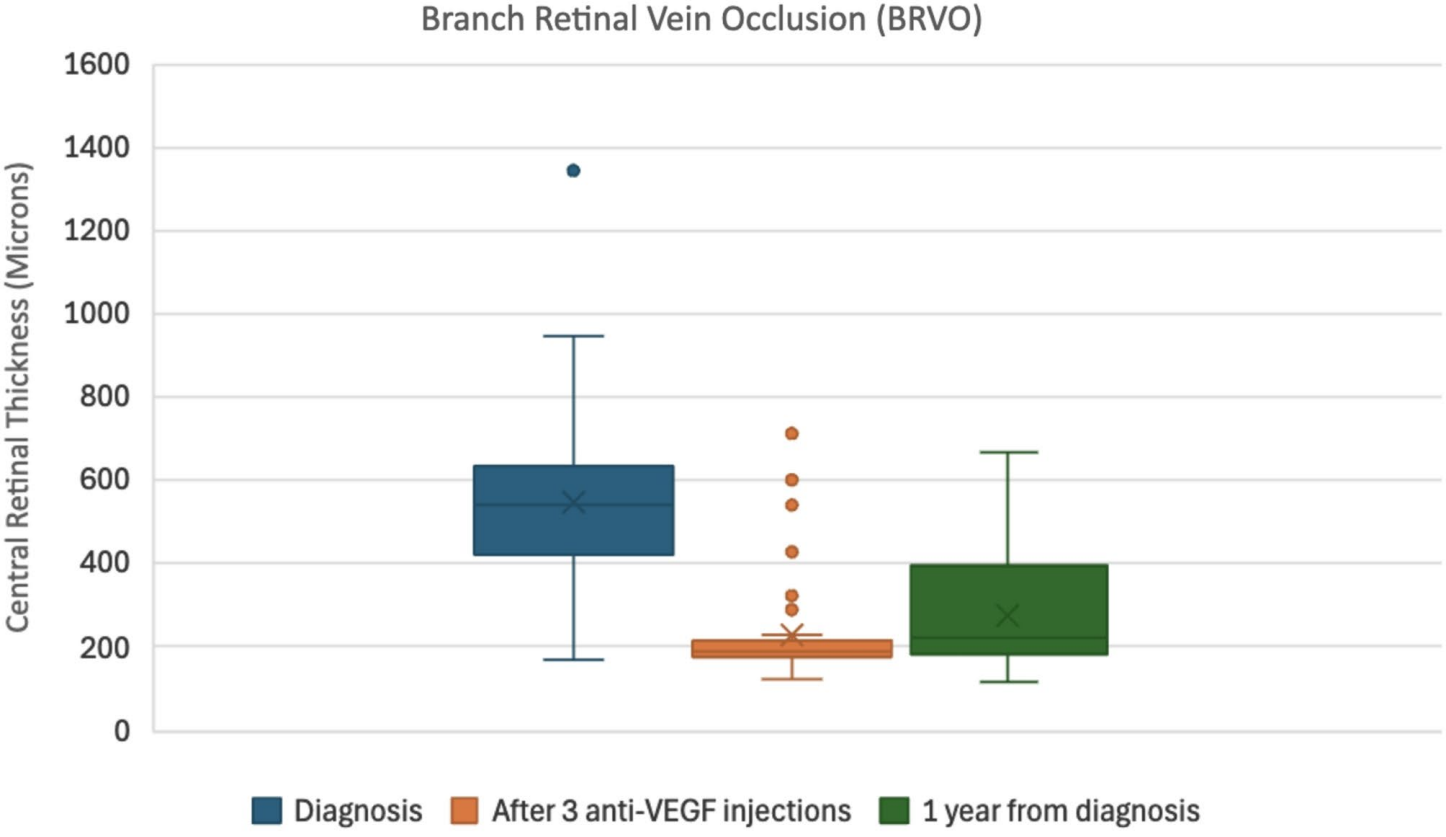
Box plot showing the median central retinal thickness in microns and inter-quartile ranges at diagnosis, after 3 loading Anti-VEGF injections, and at 1 year from diagnosis for BRVO patients

Box plots illustrating the median VA (ETDRS letters) and CRT (microns), along with interquartile ranges at diagnosis, after three loading anti-VEGF injections, and at one year for both CRVO and BRVO can be found as Figs. 1, 2, 3 and 4.

Statistical analysis using paired t-tests revealed distinct trends in visual and anatomical outcomes between CRVO and BRVO patients. For CRVO, there was a non-significant trend toward improvement in VA following the initial three anti-VEGF injections (t = − 1.80, *p* = 0.081). However, no statistically significant change was observed between baseline and the one-year mark (t = − 0.24, *p* = 0.812), and while there was a suggestion of decline between post-loading and one-year VA (t = 1.96, *p* = 0.059), it did not reach significance. In contrast, BRVO patients showed statistically significant improvements in VA both after three injections (t = − 5.14, *p* ≈ 4.73 × 10⁻⁶) and at one year (t = − 3.99, *p* ≈ 0.00023) compared to baseline. No significant change in VA was observed between post-loading and the one-year follow-up (t = 1.00, *p* ≈ 0.322), suggesting stability after the initial gains.

In terms of anatomical outcomes, both CRVO and BRVO groups demonstrated statistically significant reductions in CRT following three loading injections (CRVO: t = 6.83, *p* ≈ 2.48 × 10⁻⁷; BRVO: t = 9.47, *p* ≈ 1.78 × 10⁻¹²) and from baseline to one year (CRVO: t = 4.62, *p* ≈ 9.19 × 10⁻⁵; BRVO: t = 6.61, *p* ≈ 4.68 × 10⁻⁸). However, the changes in CRT from post-loading to the one-year mark were not statistically significant in either group (CRVO: t = − 1.38, *p* ≈ 0.179; BRVO: t = − 1.99, *p* ≈ 0.053), indicating that most anatomical improvement occurred early in the treatment course.

These results are summarised in Table 2.

**Table 2:** Paired t-test results for visual acuity (VA) and central retinal thickness (CRT) changes in CRVO and BRVO

Comparison	Outcome	Group	t-value	*p*-value	Statistically Significant
Diagnosis → After 3 injections	VA	CRVO	–1.80	0.081	No
Diagnosis → 1 year	VA	CRVO	–0.24	0.812	No
After 3 injections → 1 year	VA	CRVO	1.96	0.059	No
Diagnosis → After 3 injections	VA	BRVO	–5.14	4.73 × 10⁻⁶	Yes
Diagnosis → 1 year	VA	BRVO	–3.99	0.00023	Yes
After 3 injections → 1 year	VA	BRVO	1.00	0.322	No
Diagnosis → After 3 injections	CRT	CRVO	6.83	2.48 × 10⁻⁷	Yes
Diagnosis → 1 year	CRT	CRVO	4.62	9.19 × 10⁻⁵	Yes
After 3 injections → 1 year	CRT	CRVO	–1.38	0.179	No
Diagnosis → After 3 injections	CRT	BRVO	9.47	1.78 × 10⁻¹²	Yes
Diagnosis → 1 year	CRT	BRVO	6.61	4.68 × 10⁻⁸	Yes
After 3 injections → 1 year	CRT	BRVO	–1.99	0.053	No

## Impact of treatment timing on visual acuity and retinal thickness in CRVO and BRVO patients

### Correlation analysis

Spearman’s rank correlation was used to assess the relationship between the time from listing to first injection and both VA changes and CRT changes at different time points in CRVO and BRVO patients.

In CRVO, time to injection showed weak negative correlations with VA change after three injections (ρ = − 0.163) and at one year (ρ = − 0.081), suggesting slightly worse VA outcomes with delays. CRT reduction was weakly and positively correlated with time to injection (ρ = 0.281 at three injections; ρ = 0.109 at one year), indicating smaller CRT reductions with longer delays.

In BRVO, time to injection also showed weak negative correlations with VA change (ρ = − 0.112 at three injections; ρ = − 0.249 at one year). CRT change correlations were similarly weak (ρ = − 0.277 at three injections; ρ = − 0.650 at one year), with no consistent trend.

### Mann-Whitney U test

Treatment timing (≤ 28 days vs. >28 days) did not significantly affect VA change in CRVO at three injections (z = − 1.49, *p* = 0.136) or one year (z = − 1.04, *p* = 0.1492). However, CRT change after three injections was significantly greater with early treatment (z = − 1.673, *p* = 0.047); the difference was not significant at one year (z = − 0.463, *p* = 0.322).

In BRVO, no significant differences in VA change were found at three injections (z = − 1.218, *p* = 0.112) or one year (z = − 0.249, *p* = 0.402). CRT changes were also not significantly different at three injections (z = − 0.277, *p* = 0.391) or one year (z = − 0.650, *p* = 0.258).

### Chi-square analysis

No significant associations were found between treatment timing and likelihood of > 15-letter VA improvement, no change, or decline. In BRVO: χ²(2) = 2.57, *p* = 0.276 (three injections); χ²(2) = 1.97, *p* = 0.373 (one year). In CRVO: χ²(2) = 2.42, *p* = 0.298 (three injections); χ²(2) = 3.13, *p* = 0.209 (one year).

### Summary of findings

Timing of anti-VEGF treatment initiation (≤ 28 days vs. >28 days) did not significantly affect VA outcomes in CRVO or BRVO. In CRVO, early treatment was associated with significantly greater CRT reduction after three injections, though not at one year.

## Discussion

This study aimed to assess the impact of treatment delay on visual and anatomical outcomes in patients with newly diagnosed retinal vein occlusion (RVO) receiving anti-VEGF therapy. Our findings indicate that initiating treatment more than 28 days after diagnosis does not significantly compromise long-term VA outcomes in either BRVO or CRVO patients. These results suggest that, while timely treatment may offer some short-term anatomical benefits, long-term functional outcomes appear to be influenced by a broader range of factors beyond initial treatment timing.

Previous studies have shown that earlier anti-VEGF intervention in RVO may lead to faster resolution of macular oedema and early visual gains [4, 5]. However, our data align with more recent evidence suggesting that delayed initiation of treatment may not result in significantly poorer long-term outcomes [10, 11]. Specifically, our analysis revealed that although earlier treatment in CRVO patients was associated with a greater reduction in CRT after three injections, this effect did not persist at the one-year mark.

In addition to comparing early vs. delayed treatment groups, we conducted within-patient longitudinal analyses of VA and CRT across three timepoints—baseline, after 3 loading injections, and at one year. Paired t-tests demonstrated statistically significant improvements in VA and reductions in CRT after 3 injections in BRVO patients, with these anatomical gains largely maintained at one year. In contrast, CRVO patients showed a significant short-term reduction in CRT but no corresponding statistically significant gains in VA at either timepoint. Notably, there was a trend toward visual decline in CRVO patients between the post-loading and one-year visits, although this did not reach statistical significance (*p* = 0.059). The findings may be attributed to the more favourable natural course of BRVO compared to CRVO [12, 13]. These findings also reinforce the notion that anatomical improvements do not always translate into sustained functional benefits, particularly in more severe disease entities like CRVO. This finding is clinically relevant given current healthcare constraints and treatment backlogs. While the recommendation by RCOphth that initial evaluation and treatment for RVO should ideally occur within 2–4 weeks of presentation [7] remains a valuable target, our results indicate that a delay beyond 28 days may not uniformly compromise patient outcomes.

We also found no statistically significant link between treatment timing and categorical visual outcomes, such as the likelihood of significant VA improvement or decline. These findings may reflect the complex interplay of disease severity at presentation, individual response to treatment, adherence to follow-up, and the variability in the natural course of RVO [14, 15]. While our results suggest that initial treatment delay did not significantly affect visual outcomes at one year, it is important to acknowledge the evidence supporting the prognostic value of early intervention [16, 17]. Our findings align with this notion, particularly in the case of CRVO, where earlier treatment was associated with greater short-term anatomical improvement. However, as this benefit was not sustained at the one-year mark. Long-term outcomes may depend more on continued disease monitoring, management strategies, and patient-specific clinical factors than on the timing of the initial injection alone [16].

The finding that early versus delayed initiation of anti-VEGF therapy did not significantly influence visual acuity at one year is consistent with several real-world studies regarding treatment of retinal vein occlusion. These studies have demonstrated that visual acuity and central retinal thickness outcomes tend to converge over time, with this effect being particularly apparent in CRVO [18, 19]. This aligns with our results, as we observed a positive correlation for CRVO between early treatment and greater CRT reduction after three injections, that was not sustained at one year. Collectively, these findings suggest that while early treatment may provide short-term anatomical improvements, timing alone should not be interpreted as the primary factor determining long-term visual outcomes.

There are limitations to this retrospective study, including potential selection bias, missing data, and variability in follow-up intervals. Additionally, while the mean delay to treatment was relatively short, more extreme delays were underrepresented, limiting our ability to assess very late treatment effects. Patients who did not receive treatment were excluded, which may affect generalisability. Additionally, unmeasured confounders such as baseline ischaemia or systemic health factors may have influenced results. Although baseline visual acuity appeared similar between early and delayed treatment groups within each RVO subtype, formal adjustment for baseline VA was not performed. Baseline factors such as initial visual acuity and patient characteristics, including age and comorbidities, have been shown in prior studies to strongly influence long-term visual outcomes, suggesting that subtle imbalances could have impacted our findings [20]. Similarly, variation in the total number of anti-VEGF injections over the first year was not accounted for in our analyses. Previous research has demonstrated that treatment intensity, including the number and frequency of injections, is a key determinant of both anatomical and functional outcomes in RVO [21]. Together, these observations highlight the importance of considering baseline characteristics and treatment intensity when interpreting real-world results, and suggest that future studies should aim to explore how these factors interact with timing of therapy to influence long-term visual and anatomical outcomes.

The 28-day threshold used to define early versus delayed treatment was selected based on clinical guideline recommendations rather than a biological threshold. While this conformed to real-world service targets, it may be an oversimplification of the definition of treatment delay and obscure more subtle timing-related effects on outcomes. Using paired t-tests to compare outcomes at predefined time points may not have fully accounted for within-subject correlations across repeated measurements and may affect interpretation of longitudinal changes. A further limitation is the relatively small size of the delayed treatment group, which may have reduced statistical power for between-group and subgroup analyses and limits the strength of conclusions regarding the impact of treatment delay. Additional limitations include the retrospective design and absence of a control group, which may introduce selection bias and limit the ability to draw causal inferences regarding the effects of treatment timing. Nonetheless, the study reflects real-world practice in a tertiary centre and adds to the growing body of literature questioning the criticality of exact timing for anti-VEGF initiation in RVO.

## Conclusion

This retrospective study evaluated the impact of treatment timing on visual and anatomical outcomes in patients with BRVO and CRVO undergoing anti-VEGF therapy. Our findings suggest that initiating treatment within 28 days of diagnosis did not significantly affect long-term VA at one year in either group. Although earlier treatment in CRVO patients was associated with a greater short-term reduction in CRT after three injections, this anatomical advantage was not maintained over the longer term.

Paired analyses further revealed that while BRVO patients experienced statistically significant improvements in both VA and CRT after initial loading doses—with gains largely sustained at one year—CRVO patients showed significant anatomical improvement but no corresponding long-term visual benefit. These results underscore that early treatment may offer short-term anatomical gains, particularly in CRVO, but long-term functional outcomes appear to be influenced by additional clinical and patient-specific factors beyond the timing of the first injection.

Given existing clinical guidelines promoting early treatment initiation, these findings highlight the importance of continued disease monitoring and individualized care. They also support further research into optimizing long-term management strategies and follow-up protocols for RVO in real-world clinical settings.

## Data Availability

The datasets used and/or analysed during the current study are available from the corresponding author on reasonable request.
